# World's First Clinical Case of Gene-Activated Bone Substitute Application

**DOI:** 10.1155/2016/8648949

**Published:** 2016-11-07

**Authors:** I. Y. Bozo, R. V. Deev, A. Y. Drobyshev, A. A. Isaev, I. I. Eremin

**Affiliations:** ^1^Department of Maxillofacial and Plastic Surgery, A.I. Evdokimov Moscow State University of Medicine and Dentistry, Moscow, Russia; ^2^Human Stem Cells Institute, Moscow, Russia; ^3^Department of Maxillofacial Surgery, A.I. Burnazyan Federal Medical Biophysical Center, Moscow, Russia; ^4^I.P. Pavlov Ryazan State Medical University, Ryazan, Russia; ^5^Central Clinical Hospital with Outpatient Health Center of the Business Administration for the President of the Russian Federation, Moscow, Russia

## Abstract

Treatment of patients with large bone defects is a complex clinical problem. We have initiated the first clinical study of a gene-activated bone substitute composed of the collagen-hydroxyapatite scaffold and plasmid DNA encoding vascular endothelial growth factor. The first patient with two nonunions of previously reconstructed mandible was enrolled into the study. Scar tissues were excised; bone defects (5–14 mm) between the mandibular fragments and nonvascularized rib-bone autograft were filled in with the gene-activated bone substitute. No adverse events were observed during 12 months of follow-up. In 3 months, the average density of newly formed tissues within the implantation zone was 402.21 ± 84.40 and 447.68 ± 106.75 HU in the frontal and distal regions, respectively, which correlated with the density of spongy bone. Complete distal bone defect repair with vestibular and lingual cortical plates formation was observed in 6 and 12 months after surgery; thereby the posterior nonunion was successfully eliminated. However, there was partial resorption of the proximal edge of the autograft entailed to relapse of the anterior nonunion. Thus, the first clinical data on the safety and efficacy of the gene-activated bone substitute were obtained. Given a high complexity of the clinical situation the treatment, results might be considered as promising. NCT02293031.

## 1. Introduction

The treatment of patients with skeletal bone pathology requires frequently the use of bone substitutes, which replace the lost volumes of bone tissue and accelerate reparative osteogenesis [1]. The methods of bone grafting and the choice of bone substitute depend on the size of bone defect or bone atrophy region, coexisting disorders, and patient's age. Wide range of bone substitutes from conventional materials, such as allogenic and xenogenic bone matrix [2], hydroxyapatite [3], calcium phosphates [4], silicates [5], organic polymers [6], and their combinations, to vascularized bone autografts is available for surgery [7]. However, most of bone substitutes approved for clinical use are not effective for large bone defects repair as they could not overcome “osteogenic insufficiency” specific for such defects [8]. Therefore, physicians almost have no choice for this category of medical indications other than using bone autografts, which are characterized by a number of known limitations and drawbacks (donor site morbidity, an increased risk of complications, and a prolonged surgical intervention) [7, 8].

Different variants of “activated” bone substitutes have been developed when trying to provide an effective alternative to bone autografts [8]; these materials along with an* osteoconductive* matrix contain biological active components standardized under qualitative and quantitative parameters such as cells [9, 10], growth factors [11], or gene constructs [12, 13] which provide* osteoinduction* and/or* osteogenicity* of a bone substitute.

To date, a number of tissue-engineered bone grafts and bone substitutes with growth factors have been already approved for a clinical use. The analysis of published clinical data on their usage confirms the more efficacy compared with conventional bone substitutes [14]. However, their superiority over bone autografts remains doubtful.

Up to date, gene-activated bone substitutes have not been investigated in the clinical trials, although numerous results of experimental studies confirmed the safety and efficacy of the technological approach and individual variants of the materials [12, 13, 15–17].

We have developed a gene-activated bone substitute which consisted of two components: the collagen-hydroxyapatite scaffold and plasmid DNA encoding vascular endothelial growth factor (VEGF). This gene construct is an active substance of the drug “Neovasculgen” (PJSC Human Stem Cells Institute, Russia) which has been shown to be safe and highly effective in the treatment of patients with chronic lower limb ischemia (CLI) of stages 2a-3 and is approved for a clinical use in the territories of Russia and Ukraine [18].

The gene-activated bone substitute demonstrated an obvious osteoinductive effect in an experiment with the repair of cranial defects (with a diameter of 10 mm) in rabbits that manifested with the presence of focal reparative osteogenesis within the central defect part at 15 days after implantation. Complete restoration of bone integrity was observed in 120 days after implantation. This effect was not shown when using the same matrix without plasmid DNA: bone tissue was formed only in the periphery, from the side of bone defect edges, and there was no consolidation up to the final follow-up [19, 20].

Based on successful experimental results, we have initiated the world's first clinical trial of the gene-activated bone substitute to treat patients with maxillofacial bone defects and atrophy of alveolar ridges. The study protocol was approved by the interuniversity Ethics Committee and registered on the website https://clinicaltrials.gov/ (NCT02293031) in November of 2014.

This article describes the treatment results (safety and efficacy) for the first patient enrolled into the clinical study with 12-month follow-up.

## 2. Case Presentation

### 2.1. Patient Information

The patient, aged 37 years, a female, was admitted to the Department of Maxillofacial Surgery with complaints on the mandible mobility in the frontal and right distal regions when opening and closing the mouth and impaired mastication due to partial mandibular edentulism on the right side.

In 2011, in a clinic at place of the patient's residence, she was diagnosed to have fibrous dysplasia of the mandible on the right side, for which the patient underwent the resection of the lower jaw from the frontal region to the right ramus with external approach. Later on she had two surgeries of microsurgical mandibular reconstruction with the use of vascularized fibular bone autografts carried out in the regional clinical center. Unfortunately, the autotransplants were removed in both cases due to vascular anastomosis failure.

**Figure 1:**
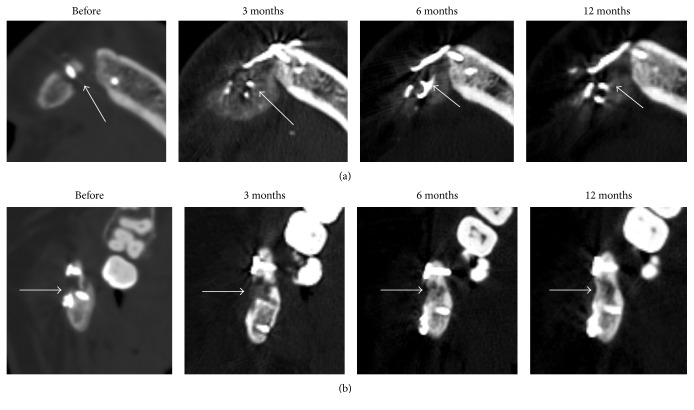
CT scans of the patient's mandible in the frontal region (a) and within the ramus on the right side (b) prior to the operation and 3, 6, and 12 months after surgery. Arrows indicate nonunions (before operation) and the sites of gene-activated bone substitute implantation.

In May 2012, a reconstruction of the right mandible was done with a free nonvascularized rib autograft in our Department. Within a year after the surgery, the patient had retention of more than 90% of the autotransplant volume and the satisfactory function of mastication on the left side. However, no consolidation was observed and nonunions were diagnosed within the proximal and distal fixation areas that caused mobility and prevented any prosthetic treatment in the mandible on the right side. Therefore, reconstructive surgery with the resection of nonunions, bone grafting, and osteosynthesis was performed. Despite the surgical intervention, a control clinical and instrumental examination in 0.5 year after the operation detected slight mobility in the frontal region and within the right ramus and no radiological evidence of consolidation (Figure 1). The diastasis in the frontal mandible ranged from 5 mm on the upper edge to 14 mm on the lower one; the average tissue density between bone edges was 158.55 ± 116.29 HU; the separation between the transplant edge and the mandibular ramus on the vestibular surface achieved 9.2 mm, with the average tissue density in the nonunion area being 204.52 ± 97.84 HU.

Taking into account a potentially poor blood supply within the fixation of the autotransplant and the mandibular fragments due to numerous operations previously performed including two failed microsurgical ones as well as a prolonged smoking experience (more than 15 years), the patient was offered to undergo a surgical treatment with the use of the gene-activated bone substitute. Considering the patient's characteristics and anamnesis, the total score according to Nonunion Scoring System [21] was estimated to be 31, which corresponded with high risk of nonunion relapse and required more specialized care. Additionally, the nonunions were complicated being formed by nonvascularized bone autograft intended to be resorbed and replaced by newly generated bone tissue. The voluntary written informed consent was obtained.

### 2.2. Gene-Activated Bone Substitute

The gene-activated bone substitute we developed consists of two components. The first one is the composite scaffold of bovine collagen and synthetic hydroxyapatite (granules with diameter of 500–1000 *μ*m) registered as a bone substitute (CJSC Polystom, Russia) and approved for clinical use in Russia, the second one is a supercoiled naked plasmid DNA with cytomegalovirus promoter and gene encoding VEGF which is the active substance of “Neovasculgen” [18]. We made the gene-activated bone substitute in the form of rectangular sponge-like matrix (size of 20 × 10 × 10 mm, weight of 200 ± 10 mg) containing 0.2 mg of the gene constructs. 5 units were used for clinical study. 5 plates were used for a surgical intervention (total amount of the scaffold: 1000 mg; total dose of the plasmid DNA: 1 mg).

### 2.3. Surgery

**Figure 2:**
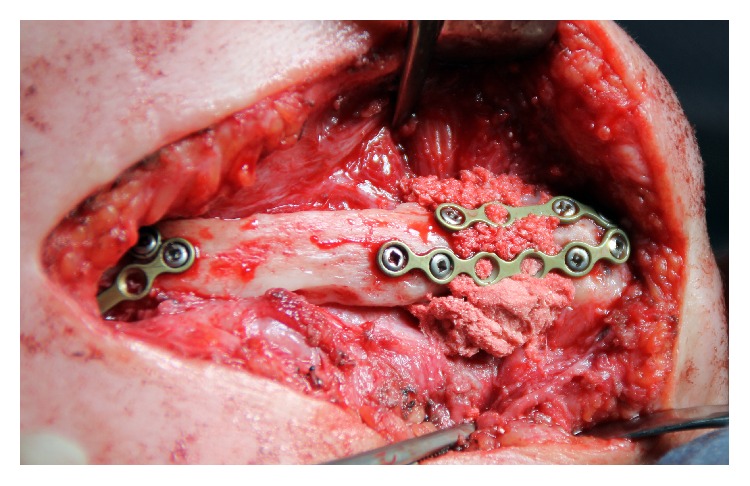
Intraoperative view: nonunions are removed; mandible fragments and rib autograft are fixed with miniplates; bone defects filled with gene-activated bone substitute.

The standard surgical protocol with metal constructs removal, nonunions fibrous tissues excision, and approximated bone surfaces careful grinding was performed. Bone defects (5–14 mm in the frontal region; 7–9 mm in the distal region of the mandible) within the rib autograft, still present from previous interventions and mandibular fragments, were filled in with the gene-activated bone substitute (Figure 2). The autotransplant was fixed in the correct position with four straight miniplates and miniscrews.

In a postoperative period, a soft diet and conservative therapy including antibiotics, analgesics, and desensitizing and anti-inflammatory agents were prescribed to the patient.

### 2.4. Safety and Efficacy Evaluation

To evaluate the treatment results, clinical and radiological diagnostic methods were used during the first 14 days of the postoperative period (in a hospital) and in 3, 6, and 12 months after surgery.

A pain level in the postoperative region was rated with the use of the Visual Analog Scale; edema was scored with the Numeric Rating Scale.

A control panoramic radiograph was made the next postoperative day; dental CT was done in 3, 6, and 12 months after surgery. A manual segmentation of the mandible was performed in the software 3D Slicer (Brigham, USA). The newly formed tissues within the bone substitute grafting were separately selected; their average density was calculated in Hounsfield units (HU) by using the “Label statistics” module. 3D bone reconstruction with volume rendering in the range of 250–2,000 HU was made, which complied with an optimal “bone window” with retention of spongy and lamellar bone in a model without metal constructs. A minimal size of diastases between mandibular fragments and rib autograft edges was determined with standard morphometry in the software Planmeca Romexis Viewer (Planmeca Oy, Finland).

### 2.5. Outcome

Neither adverse events nor serious adverse events were observed. The postoperative pain score did not exceed 6 within the first three days after surgery; it was controlled with pain-relievers; an average score for the following four days was 3.5, no pain relief was required. Later on the patient did not notice any tenderness or discomfort within the postsurgical area. The maximal edema rated as 5 by the Numeric Rating Scale was observed on the third postoperative day. Then edema gradually decreased; its score was 3 by the end of the first week and remained at the same level for up to 14 days.

**Figure 3:**
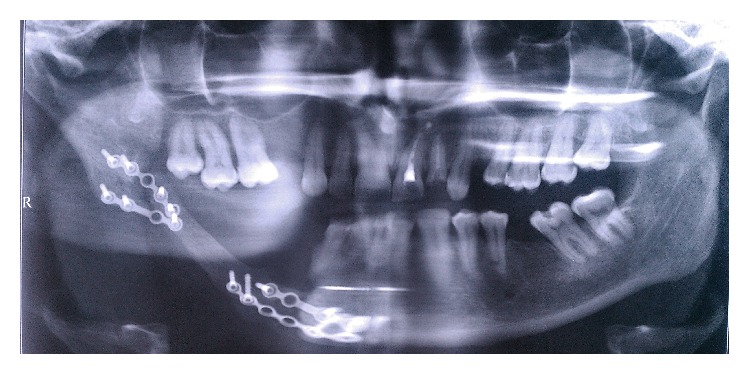
Panoramic radiography, next day after surgery.

Based on the panoramic radiograph data (Figure 3) the autograft was fixed in a right position, the gene-activated bone substitute was located within bone defects, and its radiodensity was approximately twice as less as that of the bone autograft.

No inflammation sings, edema, or pain was observed in the postsurgical area for 12 months after surgery. Control CT showed that the rib autograft and metal constructs were correctly positioned.

**Figure 4:**
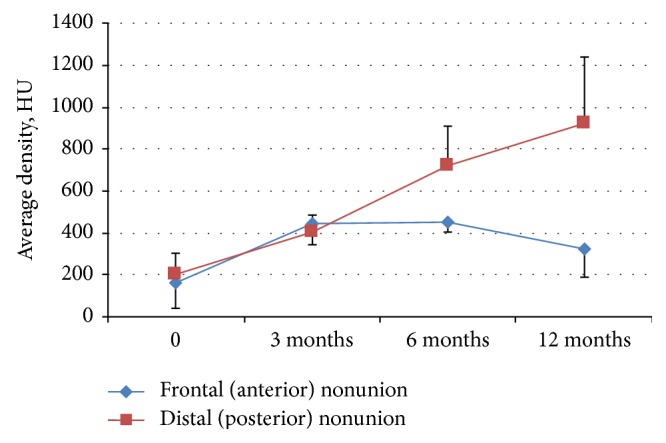
Average density in the regions of nonunions (0) and the sites of gene-activated bone substitute implantation in 3, 6, and 12 months after surgery.

3 months after surgery, increased density regions were visualized in the zones of the distal and proximal autograft fixation and bone grafting (Figure 1). The average density of these areas was 402.21 ± 84.40 in the frontal fixation and 447.68 ± 106.75 HU within the distal fixation (Figure 4).

**Figure 5:**
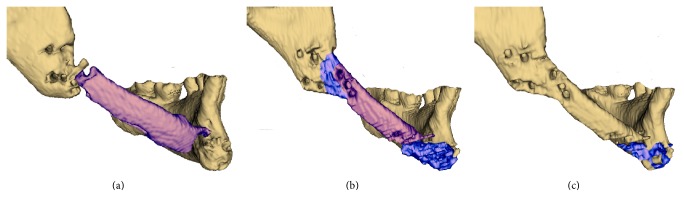
The patient's mandible in different time points after bone grafting with the gene-activated bone substitute: (a) before; (b) 6 months; (c) 12 months. Dental CT; 3D reconstruction with volume rendering 250–2000 HU (titanium constructs are excluded).

The diastasis sizes between the bone fragments were 4.8 mm on the upper edge and 12.5 mm on the lower one in the frontal surface and 6.2 mm on the vestibular surface without dissociations on the lingual one within the distal region. No defects in the zones of proximal and distal fixation of the autograft were detected using 3D reconstruction. Heteromorphic newly formed tissues were seen in these areas; the tissues overstretched the bone boarders of the reconstructed mandible outlining to a certain extent the substitute engrafted previously (Figure 5).

The newly formed tissues with average density about 400 HU within gene-activated bone substitute implantation area were observed in the frontal region 6 and 12 months after surgery (Figures 1 and 4). However, there was moderate partial resorption in the proximal edge of the bone autotransplant which prevented consolidation and maintained a diastasis. Clinical examination identified the appearance of minimal mandible mobility in the frontal region only 12 months after surgery, which corresponded with CT results.

Meanwhile, the distal edge of the rib autograft was completely integrated with adjacent mandibular ramus on both latest time points which did not allow distinguishing the borders between the mandible fragment, newly formed bone tissue, and rib autograft to segment these regions (Figure 5). Normotrophic bone callus with no defects was formed 6 months after surgery and fully mineralized later on revealing the average density of 921.51 ± 321.89 on the last time point. Moreover, we found the completed remodeling of newly formed bone tissue with distinguished vestibular (1028.67 ± 169.77 HU) and lingual (1528.78 ± 81.53) cortical plates and spongy bone between them in 12 months. No mandibular mobility was detected in this region.

## 3. Discussion

Complex clinical cases with bone defects characterized by “osteogenic insufficiency” (large ones, nonunions, etc.) [8] are the main indications for the use of activated bone substitutes as conventional ones will be suitable in less complicated settings.

Therefore, to study the safety and efficacy of the gene-activated bone substitute, we started with a very difficult clinical case when the main standard methods and materials used either resulted in specific complications or had limited efficacy. Four previous surgical interventions on the mandible resulted in scarring and impaired blood supply within the fixation areas of the bone autograft, which predisposed to the development of osteogenic insufficiency and as a consequence formation of nonunions with high score according to Nonunion Scoring System proposed by Calori et al. (2008) [21]. Moreover, one side of each nonunion was a nonvascularized bone autograft intended to be completely resorbed and simultaneously replaced by newly formed bone tissue. Such a feature has not been taken into account by current Nonunion Scoring System but obviously significantly increased a complexity of the clinical case.

Angiogenesis is known to be of critical importance for bone tissue formation [22], one of its main regulators being VEGF [23]. Moreover, in addition to affecting osteogenesis via blood vessel formation, VEGF also exerts a direct stimulating effect on cells of the osteoblastic line, enhancing their proliferation and differentiation [24]. Therefore, we used the plasmid DNA encoding* vegf* gene to make the gene-activated bone substitute. Reparative osteogenesis, as a clinical efficacy indicator, could be achieved both due to an obvious angiogenic effect of the gene construct [18–20] and owing to the direct VEGF effect on bone tissue cells.

In 3 months after surgery, newly formed tissues with an increased density equal to spongy bone were visualized within the both zones of gene-activated bone substitute grafting. The volume of those tissues correlated with that of the substitute implanted. It is known from the previous experimental studies that the gene-activated bone substitute investigated has a baseline density equal to 130 ± 350 HU. Collagen comprising 60% of the scaffold mass undergoes rapid biodegradation (within 1 month). Starting from 45 days after the operation, only single hydroxyapatite granules remain from the scaffold within the zone of a bone defect [19]. Therefore, considering a low standard deviation, bone tissue formation within the substitute implanted rather than the density of the remained scaffold fragments determined the high tissue density in the zone of bone grafting in 3 months.

The main encouraging result is that we showed that complete consolidation with normotrophic bone formation was achieved on the site of previously diagnosed distal nonunion. Bone remodeling process with cortical plates formation was completed in this area within 12 months after surgery. Unfortunately, the treatment of the other, more complex nonunion was not successful: although the gene-activated bone substitute stimulated bone formation preserved in the frontal region during the 12 months of follow-up, the partial resorption of the adjacent autotransplant edge contributed to the proximal nonunion relapse. Such a resorption of the rib bone is normal but expectedly increased in response of the surgical intervention. Basically, any nonvascularized bone autograft undergoes continuous bioresorption to be completely replaced by newly formed bone tissue. No bone substitute is able to stop this natural process. The only option for gene-activated bone substitute to provide a consolidation was to accelerate the reparative osteogenesis so highly to make it outrun the bioresorption rate. This bone formation activity was achieved in the distal nonunion site but was not enough in the proximal one. That prevents any prosthetic treatment and requires additional surgery in the frontal region with bone autografting and more rigid fixation (e.g., long custom-made titanium reconstructive plate).

Neither adverse events (hypersensitive reaction, abnormal pain, edema, inflammatory complications, development of local vascular malformations, tumors, etc.) nor serious adverse events (life-threatening conditions) were observed throughout the follow-up. Pain and edema levels in the postoperative region did not exceed the average values specific for postoperation periods of this type of surgical interventions, which confirms the substitute's safety. These clinical data on safety were expected because the gene-activated bone substitute components, the collagen-hydroxyapatite scaffold and plasmid DNA, separately were registered and approved for clinical use previously in Russia as medical devise for bone grafting and drug for CLI treatment, respectively.

Thus, considering the complexity of the case, the results of the first clinical application of the gene-activated bone substitute which is composed of the collagen-hydroxyapatite scaffold and the plasmid DNA with the* vegf *gene are quite promising and indicate the safety but limited efficacy concerning a medical indications range. Possibly, the use of more osteoconductive scaffold (e.g., xenogenic bone matrix [19]) to make a gene-activated bone substitute could extend the medical indications where it might be effective. At any case we need more clinical trials and published data to assess the treatment options and opportunities gene-activated bone substitutes could provide.
